# Prescription Departmant

**Published:** 1892-08

**Authors:** 


HAY FEVER.—By *Dr. Philpots*, France.

℞. Acid boric, pulv., 2.0 (gr. xxx.)
Salicyl. natr., 2.5 (gr. xxxviij.)
Cocaine mur., 0.12 (gr. jss.)

M. f. p. Sig. To be insuflated into the nostrils.

DIPHTHERIA.—By *Dr. Bolio*, Detroit, Mich.

℞. Quinini bi-bisulph., gr. xx.

Sulphuris, dr. j.

Yerbæ santæ syrup, oz. j.

M. S. 1-2 teaspoonful every three or four hours.

CHRONIC TONSILITIS —

℞. Acid tannic, gr. xv
Tinct. iodinii, gtt. ij.
Aquæ, f.oz. vj.
Glycerin, f. oz. ss.

M. S. Tablespoonful every three hours.

PALPITATION OF THE HEART.—By *Dr. T. A. Fairbairn*, San Diego, Cal.

℞ Tr. veratrum vir., gtt. ij.
Potass. bicarb., gr. v.
Tr. capsici, gtt. xx.

M. S. At a dose.

MENINGITIS.—By *Dr. N. S. Davis.*

℞. Potassii iodidi, dr. ijss.
Tr. digitalis, dr. iv.
Tr. hyoscyami, dr. iv.
Aquæ menthæ, oz. iij.

M. S. A teaspoonful every two or three hours, in a little sweetened water.

BRONCHIAL ASTHMA.—

℞. Iodide of ammonium, dr. 2.
Fl. ext. grindelia robusta, oz. 1-2.
Fl. ext. glycyrrhiza, dr. 4.
Tinct. lobelia, dr. 2.
Tinct. belladonna, dr. 2.
Syr. tolu, q. s. ad., oz. 4.

M. S. Teaspoonful three times a day; extra doses during a paroxysm. —*The Doctor's Weekly*

ASIATIC CHOLERA.—*Dr. R. W. Mitchell.*

℞. Acidi sulphurici, dr. ss
Morph. sulphat., gr. 1-3.
Spts. vini gallici, dr. iss.
Aquæ destillatæ, oz. iij.

M. S. Inject under the skin of the arms, legs and over the stomach, every hour, until symptoms of the disease are relieved.

Hepatic Colic.—

Lemoine uses the following methods in the treatment of hepatic colic. If no vomiting is present, he has recourse to ethereal solutions, or those containing chloroform, as follows:

R. Syrupi acaciæ, f. oz. iv.

Æther sulphur., f. oz. i.

Or

R. Chl[illegible]roform, m. xv.

Tincturæ myrrh, m. xv.

Mucilag. acaciæ, f. oz. ii.

Syrup, f. oz. iis.

A tablespoonful of either of these prescriptions every fifteen minutes. —*Review Therapeutique.*

Cholera Morbus of Malarial Fever.—By *Dr. A. B. Palmer.*

R. Opium, gr. ij.
Camphor gum, gr. ij.
Calomel, gr. iij.
Sugar milk, gr xv.

M S. Rub up into a very fine impalpable powder. This should be dropped into a teaspoonful of water, and taken far back in the mouth, followed by a single small swallow of water. Repeat in one hour, if relief does not follow.

Fibroid Phthisis.—*Dr. A. B. Palmer.*

R. Iodide of potassium, dr. v.
Syrup of tolu, oz. ij.
Glycerine, oz. ij.
Syrup of ipecac, oz i.
Fl. ext. of veratrum vir , dr. i.
Sulphat. morphine, gr. iij.

M. S. A teaspoonful three to four times daily.

Laryngeal Tuberculosis —

*Dr. Cozzolino* recommends, in the *Revista de las Hospitales*, the following solutions, as spray:

R. Aquæ dest., oz. ix., dr. v.
Alcohol rectif., dr. iiss., dr. iij.
Menthol, gr. viiss., gr. xiiss
Balsam peruv. scruple iv., dr. iiss

Anemia —*Dr. T. Milner Fothergill.*

R. Arsenic, gr. i.
Ferri sulph. exiccat., dr. ss.
Pulv. pip. nig., dr. i.
Pil. al. et myrrh, dr. i.

M. Et ft. pil. No. XL. Sig One twice daily, after meals.

